# Management of Complex Facial Injuries: Cutting Traumas by Angle Grinders

**DOI:** 10.1155/2020/1891250

**Published:** 2020-05-17

**Authors:** Holger Sudhoff, Jan Schulte am Esch, Nazli Ay, Ingo Todt

**Affiliations:** ^1^Department of Otolaryngology, Head and Neck Surgery, Klinikum Bielefeld, Bielefeld, Germany; ^2^Department of General and Visceral Surgery, Protestant Hospital of Bethel Foundation, Bielefeld, Germany

## Abstract

The use of angle grinders can lead to complex facial injuries. The most frequent sites affected are within the head and face region. Anatomical boundaries or structures are not respected by the high-speed disc of angle grinders, and thus, injuries can be mutilating, permanently disabling, or even lethal. Functional and aesthetical satisfying results can be reached through debridement, excision of wound edges, and meticulous layered functional closure after appropriate reconstruction of additional facial bony defects. The management and short-term outcome of a complex facial cutting trauma by an angle grinder are presented and discussed.

## 1. Introduction

Penetrating injuries are infrequent but expose a variable entity of craniofacial traumas for the management by otorhinolaryngology, head and neck, maxillofacial, or plastic surgery. The close anatomical relationships of vital structures stress the distinctive importance to the primary clinical evaluation and management of such patients. Facial injuries resulting from the use of angle grinders are common. Nevertheless, most cases are succeeding to penetrating injuries by split-offs originating from the angle grinder's working surface or from its cutting disc. Only on rare circumstances, severe trauma results from direct cutting injury by the working tool. Here, we present two cases of a complex trauma to the facial midline due to out-of-control angle grinders.

## 2. Case Reports

**Figure 1:**
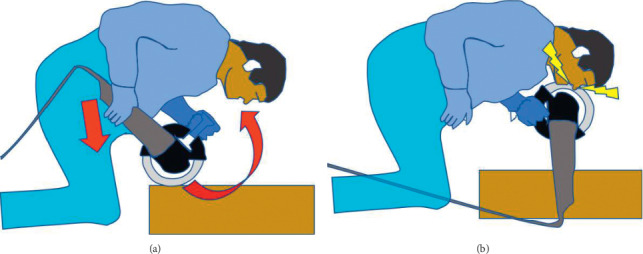
Schematic drafts highlighting the mechanism of the described angled grinder injury.

**Figure 2:**
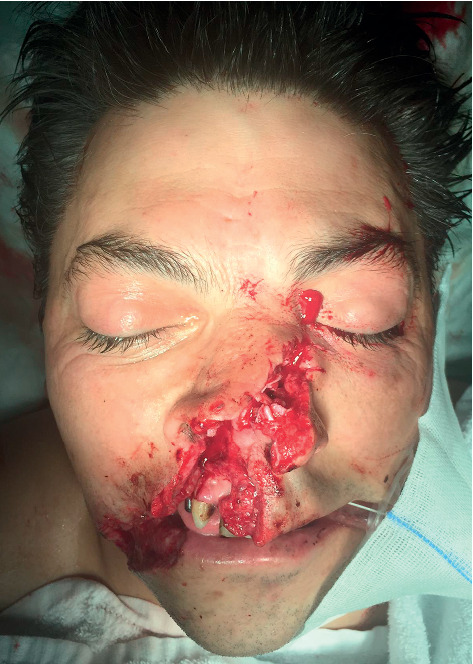
Preoperative appearance approximately one hour after the initial trauma.

**Figure 3:**
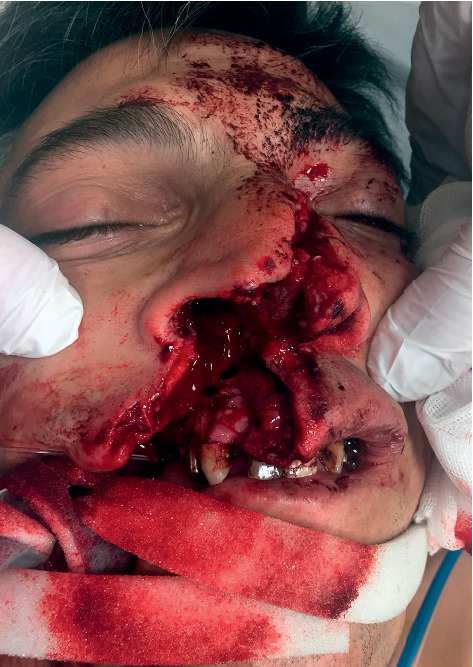
Initial facial wounds pulled apart to visualize and remove fragmented bony pieces and the depth of the grinder injury.

**Figure 4:**
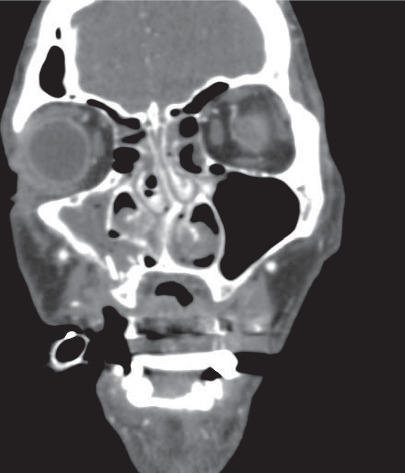
Preoperative coronal computed tomography showing a right anterior inferior maxillary sinus fracture with a hematoma in the right maxillary sinus.

**Figure 5:**
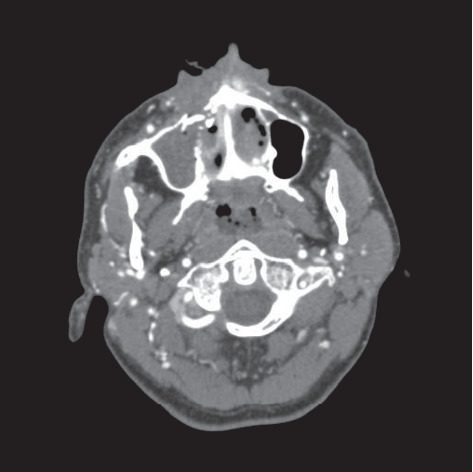
Preoperative axial computed tomography showing a right anterior fracture line of the maxillary sinus associated with hematoma and a small scattered radio-opaque fragment.

**Figure 6:**
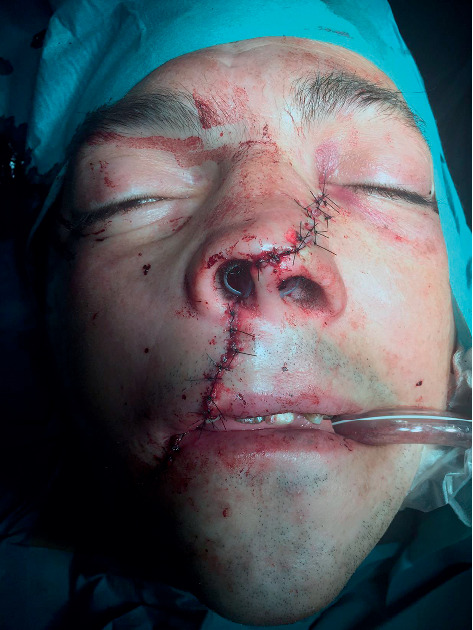
Postoperative appearance immediately after the end of the surgical procedure. Doyle open lumen splint was used to keep the nasal airways patent while maintaining support of the fractured nasal septum.

**Figure 7:**
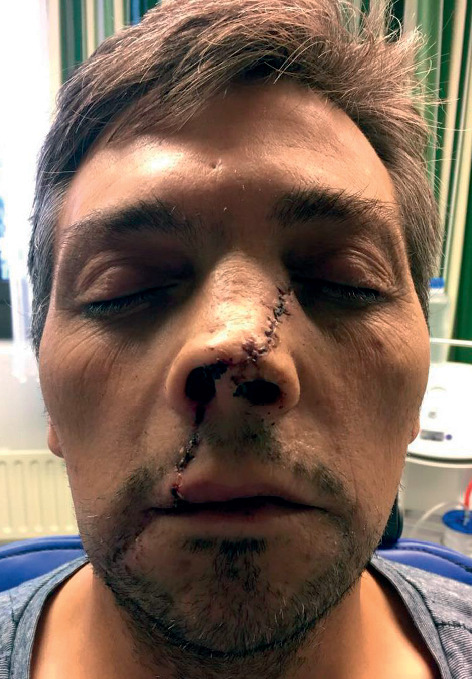
Postoperative appearance at 1 week with suture material in place.

**Figure 8:**
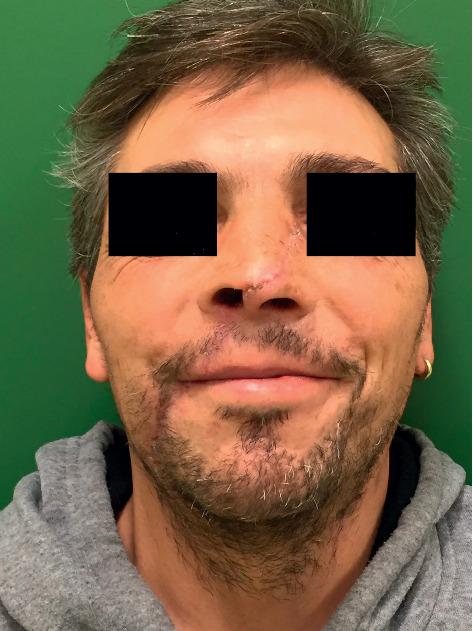
Very satisfactory postoperative outcome 2 weeks after the trauma.

**Figure 9:**
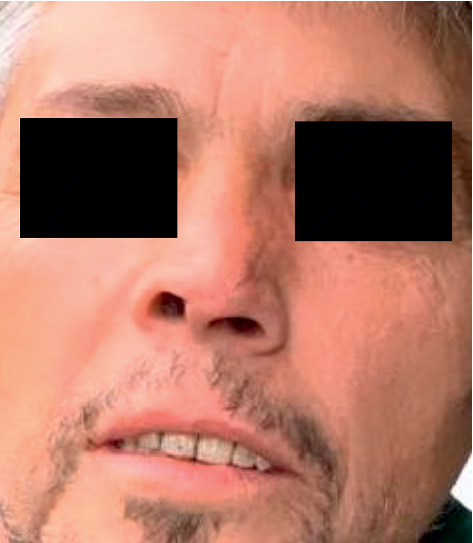
Very satisfactory postoperative outcome 1 year after the trauma.

Case 1: a 46-year-old Caucasian construction site worker was admitted intubated to the emergency room with a penetrating injury of an angle grinder without a guard. In the attempt to loosen an angle grinder fixed within a notch, the power cut suddenly got detached (Figure 1). Rebounding in a turning manner with the exposed unprotected area of the tool to the midface line, the spinning cutting disc caused the complex trauma (Figures 2 and 3). Facial nerve function was intact, and no neurological deficit was detected prior to intubation. The initial computed tomography showed the left nasal bone as well as the right anterior frontal and maxillary sinus fractures with a hematoma in the right maxillary sinus and small scattered radio-opaque fragments (Figures 4 and 5). After the reposition of the displaced nasal bony fragments and metallic foreign body material, excision of wound edges was followed by meticulous layered functional closure starting with vermilion. Doyle splints were used to keep the nasal airways patent while maintaining support of the cartilaginous septal fracture for one week (Figure 6). The postoperative appearance of the severely injured one week and two weeks after surgery was very satisfactory with the functional and aesthetical outcome (Figures 7–9).

**Figure 10:**
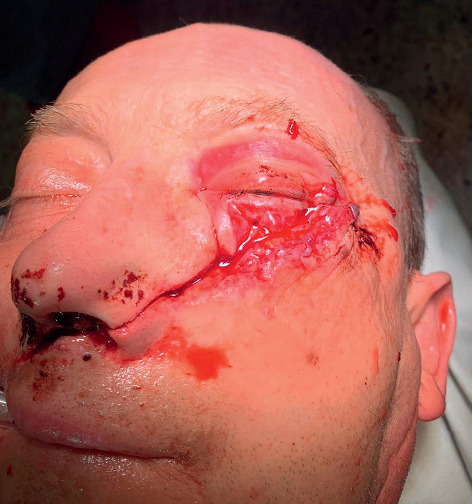
Preoperative gross appearance approximately three hours after the initial trauma.

**Figure 11:**
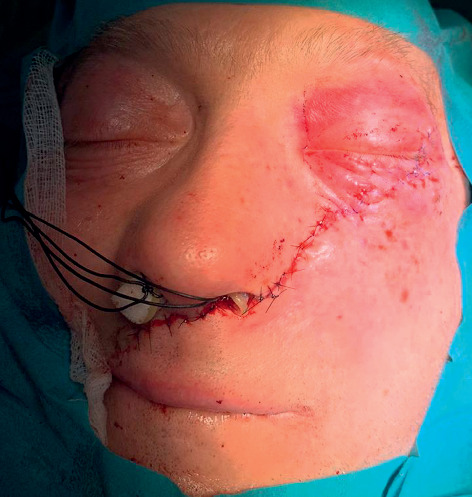
Postoperative appearance after the surgical procedure.

Case 2: a 68-year-old Caucasian hobby worker was admitted to the emergency room with a penetrating injury of an angle grinder again without a guard. In the attempt to cut through a steel pipe, the power cut suddenly flipped back. This led to a complex midface trauma (Figure 10). This less severe injury required as well as a layered functional closure of the wound including debridement of contaminated wounds and excision of ragged edges leading to an acceptable postoperative appearance (Figure 11) and a regular postoperative outcome. Oral mucosal and muscle layer closure was accomplished using Vicryl® resorbable, skin by nonresorbable monofilament sutures. Both patients received amoxicillin and clavulanate treatment adopted to their body weight.

## 3. Discussion

Angle grinders are used worldwide in high frequencies to cut hard or abrasive materials such as stone, ceramics, metal, or concrete [1]. Developed originally as a tool for rigid abrasive discs, the availability of an interchangeable power source has facilitated their use with a widespread variety of cutters and attachments. Angle grinder injuries are a source of serious morbidity and mortality, much of which are preventable. Injury mechanisms are related to direct or indirect traumas or noise-induced hearing loss. Sound pressure level and vibration study have concluded that grinders ranged from 91 to 103 dB SPL (decibel sound pressure level). Maximum peak levels at the disc reached 140 dB SPL. Most noise was caused by metal-to-metal contact, compressed air, equipment vibration, or operation of grinders [2]. The discs themselves rotate between 6,000 and 15,000 revolutions per minute, depending on the machine type and the disc diameter used. In addition to facial injuries, major injuries are frequently confined to the upper limbs and, less commonly, the lower trunk [1, 2].

The gross appearance of the wounds induced by angle grinders generally follows the shape of the cutting disc. However, it may vary slightly depending on the angulation of the skin penetration. Tissue volume loss is commonly seen. It is dependent on the size of the disc. Finding fragments of the disc and the material being cut in the wound is a typical observation in angle grinder injuries [3]. Consequently, a comprehensive debridement of contaminated wounds and excision of ragged edges are necessary to ensure optimal healing. Especially, case 1 includes a severe nasal injury that might lead to nasal vestibular stenosis or alar retraction. Doyle splints should be considered to support transcartilaginous mattress sutures in order to prevent displacement and ensure stabilization by internal splinting.

Injuries can occur for multiple reasons. The disc itself may kick back from working while cutting as it happened in our two cases. This will push the rotating disc parallel towards the patient. Therefore, the facial region is most often affected by penetrating wounds [4]. This clinical presentation is found in all cases reported as all reveal oblique or parasagittal lacerations parallel to the cutting axis. An additional reason is the use of the wrong or a damaged disc. This will enlarge the probability of excessive vibrations and disc shattering. This can lead to a foreign body-type injury requiring antibiotic treatment. A thorough clinical examination should be performed in the situation of a shattered disc as several anatomical locations may be affected [2]. Lethal intracranial injuries have been reported with the overhead use of angle grinders and should be strictly avoided [5]. The risk of injury is significantly reduced by paying attention to the general guidelines about the use of angled grinders and on the use of protective guards and suitable clothing [1]. Both cases show that satisfying results can be accomplished by meticulous debridement, excision of wound edges, and layered functional closure after appropriate reconstruction. Dependent on severity of the grinder injury, extended facial bony defects may require osteosynthesis techniques using, e.g., titanium microplates. Using vermilion to start the skin closure was recommendable in these cases to obtain satisfying results. The presented cases exemplify that the high-speed disc of angle grinders does not follow anatomical structures and can be extremely disfiguring.

## Data Availability

All relevant data are reported in the manuscript.
